# Antiandrogenic Effects of a Polyphenol in *Carex kobomugi* through Inhibition of Androgen Synthetic Pathway and Downregulation of Androgen Receptor in Prostate Cancer Cell Lines

**DOI:** 10.3390/ijms232214356

**Published:** 2022-11-18

**Authors:** Yudai Kudo, Satoshi Endo, Masatoshi Tanio, Tomofumi Saka, Rin Himura, Naohito Abe, Mitsumi Takeda, Eiji Yamaguchi, Yuta Yoshino, Yuki Arai, Hirohito Kashiwagi, Masayoshi Oyama, Akichika Itoh, Masaki Shiota, Naohiro Fujimoto, Akira Ikari

**Affiliations:** 1 Laboratory of Biochemistry, Gifu Pharmaceutical University, Gifu 501-1196, Japan; 2 Laboratory of Pharmacognosy, Gifu Pharmaceutical University, Gifu 501-1196, Japan; 3 Laboratory of Pharmaceutical Synthetic Chemistry, Gifu Pharmaceutical University, Gifu 501-1196, Japan; 4 Universal Corporation Co., Ltd., Gifu 502-0931, Japan; 5 Department of Urology, Graduate School of Medical Sciences, Kyushu University, Fukuoka 812-8582, Japan; 6 Department of Urology, University of Occupational and Environmental Health, Kitakyushu 807-8555, Japan

**Keywords:** androgen receptor, DHRS11, prostate cancer, polyphenol, *Carex kobomugi*

## Abstract

Prostate cancer (PC) represents the most common cancer disease in men. Since high levels of androgens increase the risk of PC, androgen deprivation therapy is the primary treatment; however this leads to castration-resistant PC (CRPC) with a poor prognosis. The progression to CRPC involves ectopic androgen production in the adrenal glands and abnormal activation of androgen signaling due to mutations and/or amplification of the androgen receptor (AR) as well as activation of androgen-independent proliferative pathways. Recent studies have shown that adrenal-derived 11-oxygenated androgens (11-ketotestosterone and 11-ketodihydrotestosterone) with potencies equivalent to those of traditional androgens (testosterone and dihydrotestosterone) are biomarkers of CRPC. Additionally, dehydrogenase/reductase SDR family member 11 (DHRS11) has been reported to be a 17β-hydroxysteroid dehydrogenase that catalyzes the production of the 11-oxygenated and traditional androgens. This study was conducted to evaluate the pathophysiological roles of DHRS11 in PC using three LNCaP, C4-2 and 22Rv1 cell lines. DHRS11 silencing and inhibition resulted in suppression of the androgen-induced expression of AR downstream genes and decreases in the expression of nuclear AR and the proliferation marker Ki67, suggesting that DHRS11 is involved in androgen-dependent PC cell proliferation. We found that 5,7-dihydroxy-8-methyl-2-[2-(4-hydroxyphenyl)ethenyl]-4*H*-1-benzopyran-4-one (Kobochromone A, KC-A), an ingredient in the flowers of *Carex kobomugi,* is a novel potent DHRS11 inhibitor (IC_50_ = 0.35 μM). Additionally, KC-A itself decreased the AR expression in PC cells. Therefore, KC-A suppresses the androgen signaling in PC cells through both DHRS11 inhibition and AR downregulation. Furthermore, KC-A enhanced the anticancer activity of abiraterone, a CRPC drug, suggesting that it may be a potential candidate for the development of drugs for the prevention and treatment of CRPC.

## 1. Introduction

Prostate cancer (PC) represents the most common cancer disease in men. Androgen deprivation therapy (ADT) is the primary treatment for PC, because localized and metastatic PC grows in an androgen-dependent manner [1,2,3]. ADT can provide remission in the early stages of treatment, and in many cases the cancer relapses within six months to several years, leading to castration-resistant PC (CRPC) with a poor prognosis [4,5,6]. Although ADT controls the production of androgens in the testis, it cannot reduce adrenal androgen synthesis [7,8,9,10]. The causes of the progression to CRPC include ectopic androgen production in adrenal glands and abnormal activation of androgen signaling due to mutations and/or amplification of the androgen receptor (AR), as well as aberrant activation of androgen-independent signaling such as the phosphatidylinositol 3-kinase/Akt/mammalian target of rapamycin signaling [11,12].

**Figure 1:**
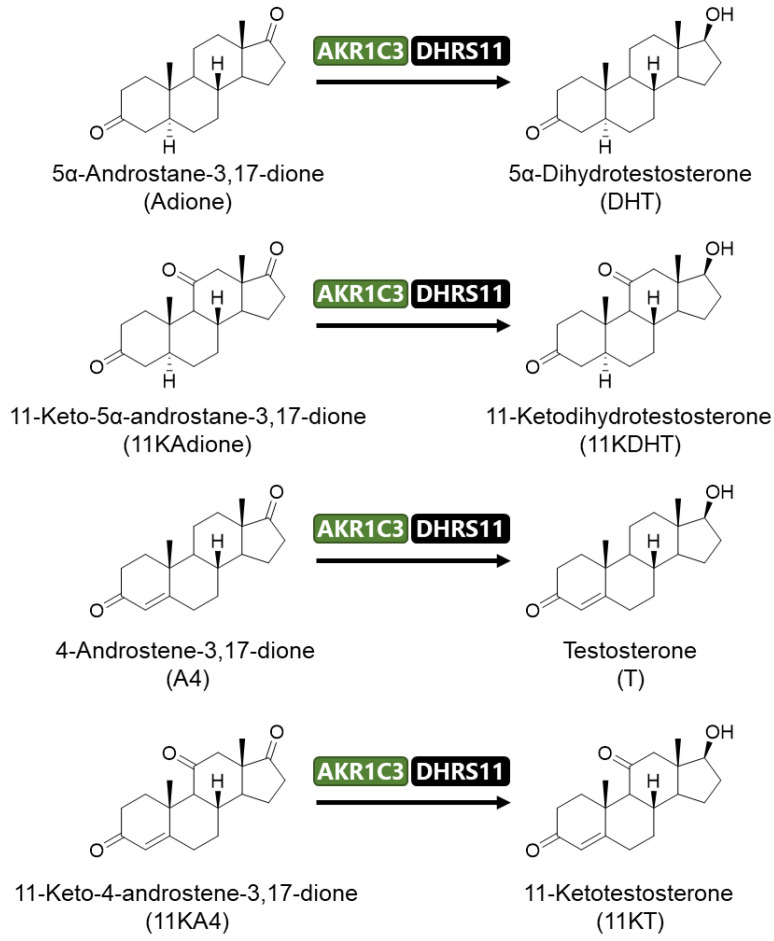
Representative reactions catalyzed by AKR1C3 and DHRS11. The two enzymes catalyze the synthesis of the potent androgens (DHT, 11KDHT, T and 11KT) via their 17βHSD activity.

In the adrenal gland, 4-androstene-3,17-dione (A4) and 5-androstene-3β,17β-diol are produced from the androgen precursor dehydroepiandrosterone (DHEA) and its sulfate (DHEAS), which are converted to the potent androgen testosterone in target tissues such as the testis [13,14,15]. Testosterone (T) is converted to 5α-dihydrotestosterone (DHT), the most potent androgen, by 5α-reductase. Recent studies have shown that 11-oxygenated androgen precursors, such as 11β-hydroxy-4-androstene-3,17-dione (11OHA4) and 11β-hydroxytestosterone, are also produced in the adrenal gland [16]. 11OHA4 is oxidized to 11-keto-4-androstene-3,17-dione (11KA4) by 11β-hydroxysteroid dehydrogenase type-2 and metabolized to 11-keto-5α-androstane-3,17-dione (11KAdione) by 5α-reductase. 11KA4 and 11KAdione are converted to the potent androgens 11-ketotestosterone (11KT) and 11-ketodihydrotestosterone (11KDHT), respectively, by 17β-hydroxysteroid dehydrogenases (17βHSDs). 11KT and 11KDHT have androgenic effects comparable to the classical androgens T and DHT [17,18,19]. Therefore, much attention has been paid to the biological roles of 11-oxygenated androgens derived from the adrenal glands in the development of CRPC [17,20,21,22], and the importance of controlling their production from a therapeutic perspective. In fact, abiraterone acetate (Abi), which is used as a CRPC drug, decreases the level of 11-oxygenated androgens through the inhibition of cytochrome P450 17-hydroxylase/17,20-lyase (CYP17A1) activity [23]. Recently, we found that, in addition to aldo-keto reductase (AKR)1C3 (also known as 17βHSD5) and 17βHSD3, human dehydrogenase/reductase SDR family member 11 (DHRS11) is involved in the production of T, DHT, 11KT and 11KDHT as a 17βHSD [24] (Figure 1). DHRS11 and AKR1C3 show the same catalytic efficiencies for the 11-oxygenated androgen precursors (11KA4 and 11KAdione), although such kinetic constants have not been determined for 17βHSD3. The differences in the expression levels and pathophysiological effects of these 17βHSDs in PC cell lines remain unclear.

To clarify the pathophysiological roles of DHRS11 and its association with PC, we have evaluated the effects of DHRS11 knockdown and inhibition on androgen synthesis and androgen signaling in three PC cell lines (LNCaP, C4-2 and 22Rv1). We, for the first time, have searched for novel functional molecules from *Carex kobomugi* flowers and identified 5,7-dihydroxy-8-methyl-2-[2-(4-hydroxyphenyl)ethenyl]-4*H*-1-benzopyran-4-one (Kobochromone A, KC-A) as a novel potent DHRS11 inhibitor. Moreover, we examined the effects of KC-A on the AR expression and androgen-dependent proliferation of PC cells.

## 2. Results and Discussion

### 2.1. Activation of Androgen Signaling by 11-Oxygenated Androgens

**Figure 2:**
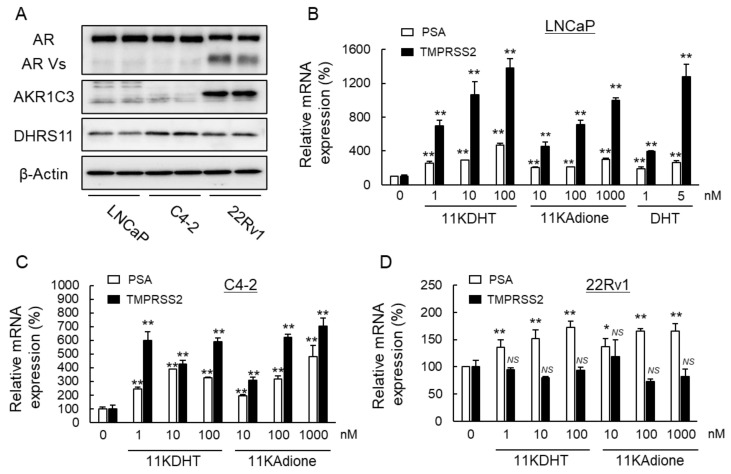
Differences in the expression of 17βHSDs and androgen sensitivity among three PC cell lines (LNCaP, C4-2 and 22Rv1). (**A**) Expression of AR, AKR1C3 and DHRS11 were detected by immunoblotting. (**B**–**D**) Effects of 11-ketoandrogens on androgen signaling in LNCaP (**B**), C4-2 (**C**) and 22Rv1 (**D**) cells. The cells were treated for 24 h with the indicated concentrations of 11KDHT, 11KAdione or DHT. The mRNA expression levels of PSA and TMPRSS2 in the cells were analyzed by RT-qPCR analysis. ** *p* < 0.01, * *p* < 0.05, *NS p* > 0.05 compared with the control cells treated with DMSO alone.

AKR1C3 is overexpressed in PC tissues and cell lines [25], but the expression of DHRS11 in PC tissues has not been reported, although its mRNA is expressed in human prostate and several cancer cell lines such as A172, MCF7, U937 and C32TG cells derived from human glioblastoma, breast adenocarcinoma, lymphoma and melanoma [26]. We first examined the expressions of the two enzymes, together with AR and its variants (AR Vs), in three PC cell lines: LNCaP, C4-2 and 22Rv1 (Figure 2A). AKR1C3 was highly expressed in 22Rv1 cells, whereas DHRS11 was expressed in all the PC cell lines. Wild-type AR was expressed in the three PC cell lines, and its variants were detected only in that of 22Rv1 cells as previously reported [27].

In the absence of androgens, AR forms an inactive complex with heat shock proteins and localizes to the cytoplasm [28]. When the ligand androgen binds, AR is dissociated from the heat shock protein and translocated into the nucleus, where it binds to androgen-responsive sequences in the regulatory regions of target genes and promotes cell proliferation. The target genes include prostate specific antigen (PSA) and transmembrane protease serine 2 (TMPRSS2). Recently, Pretorius et al. [17] reported that 11KDHT and 11KT exhibit androgenic activity comparable to DHT and T in LNCaP and VCaP cell lines. However, the effect of 11KAdione, a precursor of 11KDHT, on androgen signaling has not been investigated. Therefore, we compared the effects of 11KDHT and 11KAdione on androgen signaling in the three LNCaP, C4-2 and 22Rv1 cells by analyzing the expression of mRNAs for PSA and TMPRSS2. In androgen-sensitive LNCaP cells, 11KDHT and 11KAdione, as well as DHT, markedly induced the expression of PSA and TMPRSS2 (Figure 2B). A similar induction of the AR-target genes by 11KDHT and 11KAdione was observed in C4-2 cells, which are known as a partially androgen-sensitive PC cells [29] (Figure 2C). In contrast, the levels of PSA induction by 11KDHT and 11KAdione were low and no induction of TMPRSS2 was observed in 22Rv1 cells, which are defined as ectopic androgen-insensitive CRPC cells (Figure 2D). The activation of androgen signaling by 11KAdione was observed at concentrations more than 10-fold higher than that by 11KDHT in LNCaP and C4-2 cells, indicating that 11KAdione is metabolized to 11KDHT. The results suggest that DHRS11 and/or AKR1C3, which have 17βHSD activity converting 11KAdione to 11KDHT, are important factors for the activation of androgen signaling in these cells.

### 2.2. Effect of DHRS11 Silencing on 11KAdione-Induced Androgen Signaling

**Figure 3:**
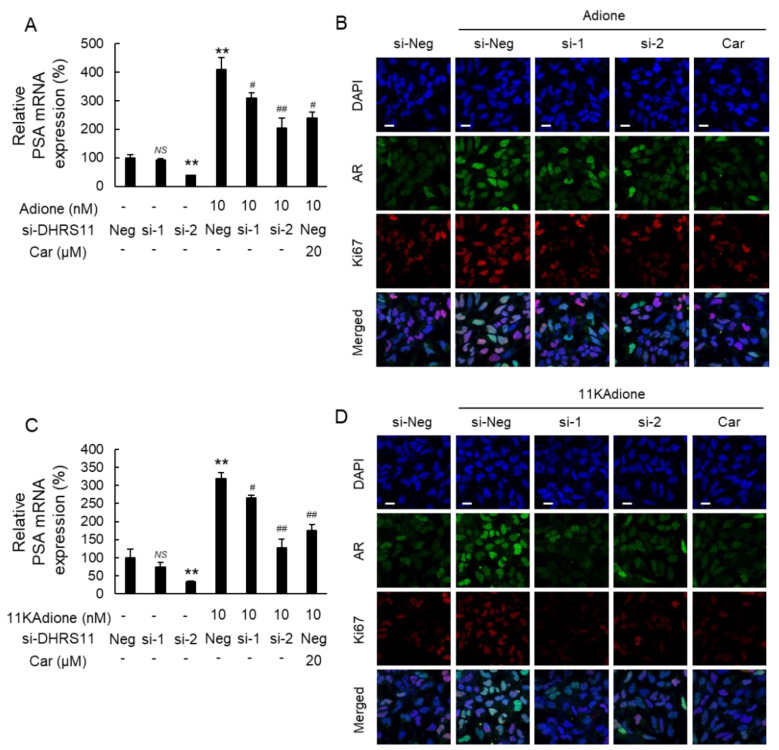
Effects of DHRS11 knockdown on androgen signaling induced by Adione or 11KAdione in C4-2 cells. The cells were treated with Adione (**A**,**B**) or 11KAdione (**C**,**D**) 24 h after transfection with DHRS11 siRNA (si-1 or si-2) or universal negative siRNA (Neg) and incubated for another 24 h. Treatment with a DHRS11 inhibitor, carbenoxolone (Car), was performed 2 h before the treatment with Adione or 11KAdione. (**A**,**C**) PSA expression. The expression levels analyzed by RT-qPCR. ** *p* < 0.01, *NS p* > 0.05 denotes significantly different from the cells treated with DMSO alone. ^##^ *p* < 0.01, ^#^ *p* < 0.05 denotes significantly different from the cells treated with 10 nM Adione or 11KAdione alone. (**B**,**D**) Nuclear expression of AR and Ki67. AR (green), Ki67 (red) and DNA (blue) were visualized by immunofluorescence using the antibodies against AR and Ki67 and DAPI, respectively. Merged images are also shown. Scale bar indicates 20 μm.

Solid tumors, including PC cells, are known to be a heterogeneous cell population, and PC cells contain cell populations with different androgen sensitivity. In fact, the expression levels of AKR1C3 were apparently different among the three PC cells (Figure 2A). Although the expression levels of DHRS11 in clinical samples need to be investigated in the future, the universal expression of DHRS11 in the presently used PC cells might indicate the usefulness of DHRS11 inhibitors in the treatment of PC. To clarify whether DHRS11 is involved in the reduction of Adione and 11KAdione to DHT and 11KDHT, respectively, we investigated the effects of DHRS11 siRNA and carbenoxolone, a known inhibitor of DHRS11 [26], on androgen signaling in C4-2 cells (Figure 3). The two siRNAs (si-1 and si-2) reduced DHRS11 mRNA expression by about 80% (Figure S1) and significantly suppressed PSA expression induced by 10 nM Adione or 11KAdione (Figure 3A,C). Immunofluorescence staining revealed that DHRS11 silencing inhibited the increased expression of nuclear AR and the proliferation marker Ki67 induced by Adione or 11KAdione (Figure 3B,D), and similar results were obtained when carbenoxolone was treated. These data demonstrate the involvement of DHRS11 in the synthesis of DHT and 11KDHT in C4-2 cells.

**Figure 4:**
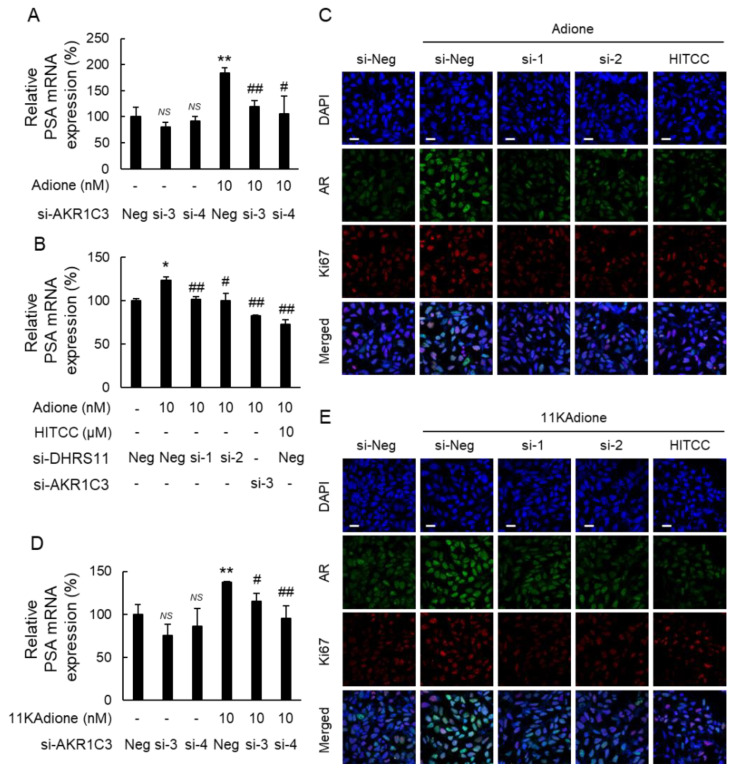
Effects of AKR1C3 knockdown on androgen signaling induced by Adione or 11KAdione in 22Rv1 cells. The cells were treated with Adione (**A**–**C**) or 11KAdione (**D**,**E**) 24 h after transfection with AKR1C3 siRNA (si-3 or si-4), DHRS11 siRNA (si-1, si-2) or universal negative siRNA (Neg) and incubated for another 24 h. Treatment with the AKR1C3 inhibitor, HITCC, was performed 2 h before the treatment with Adione. (**A**,**B**,**D**) PSA expression. The mRNA expression levels of PSA analyzed by RT-qPCR. ** *p* < 0.01, * *p* < 0.05, *NS p* > 0.05 denotes significantly different from the cells treated with DMSO alone. ^##^ *p* < 0.01, ^#^ *p* < 0.05 denotes significantly different from the cells treated with 10 nM Adione or 11KAdione alone. (**C**,**E**) Nuclear expression of AR (green), Ki67 (red) and DNA (blue) were visualized by immunofluorescence using the antibodies against AR and Ki67 and DAPI, respectively. Merged images are also shown. Scale bar indicates 20 μm.

Next, we examined the role of AKR1C3 in the reduction of Adione and 11KAdione using 22Rv1 cells, which express high levels of AKR1C3 compared to DHRS11 (Figure 2A). AKR1C3 knockdown significantly suppressed PSA expression induced by Adione or 11KAdione and inhibited nuclear AR and Ki67 expression (Figure 4). AKR1C3 has attracted attention as a promising target of CRPC and the development of AKR1C3 inhibitors has been intensified. So far, we reported that baccharin in Brazilian green propolis and its derivatives inhibit PC cell growth via AKR1C3 inhibition [30,31], and subsequently discovered a potent inhibitor of AKR1C3, 8-hydroxy-2-imino-*N*-(*p*-tolyl)-2*H*-chromene-3-carboxamide (HITCC), which inhibits PC cell growth through both androgen-dependent and androgen-independent mechanisms [29]. The treatment of cells with HITCC significantly suppressed Adione-induced PSA expression (Figure 4B). Although the amount of DHRS11 was lower than that of AKR1C3 in 22Rv1 cells (Figure 2A), DHRS11 knockdown also significantly decreased Adione-induced PSA expression in this cell (Figure 4B), in which both AKR1C3 and DHRS11 may function as 17βHSDs in androgen synthesis. Since DHRS11 silencing or inhibition suppressed androgen signaling in these three PC cells, DHRS11 inhibitors could also be candidates for the treatment of PC.

### 2.3. Suppression of Androgen Signaling Induced by Traditional Androgens and 11-Oxygenated Androgens by Novel Polyphenol KC-A in Carex Kobomugi

**Figure 5:**
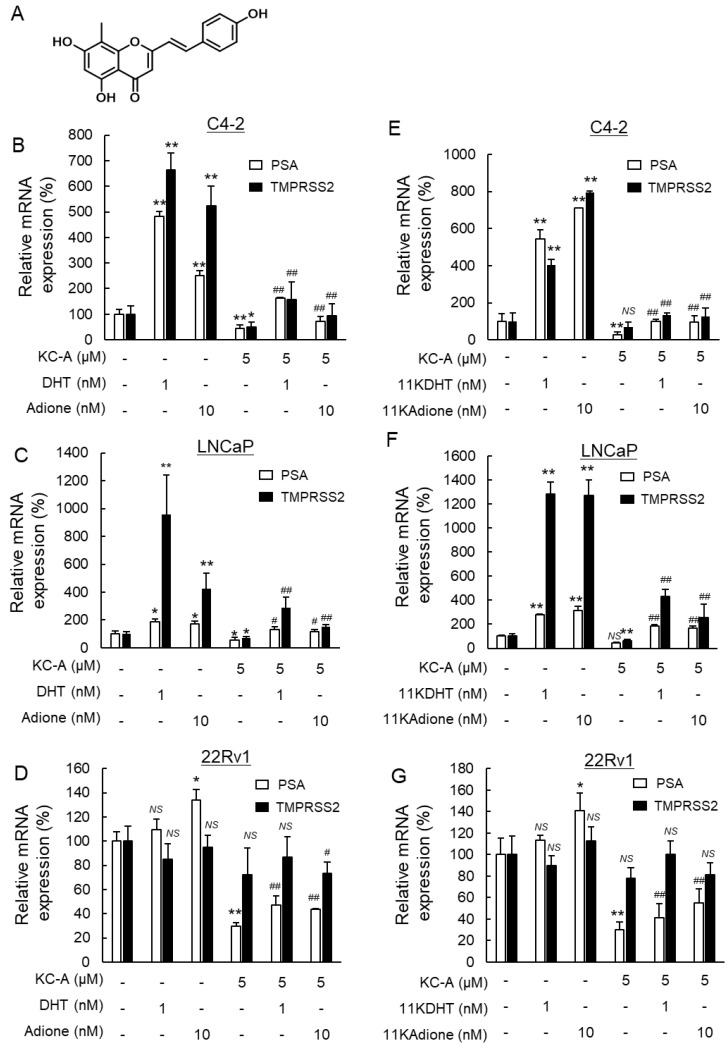
Structure of KC-A (**A**) and its effects on classic androgens and 11-keto-androgens regarding the induction of androgen signaling in C4-2 (**B**,**E**), LNCaP (**C**,**F**) and 22Rv1 (**D**,**G**) cells. The cells were pretreated for 2 h without or with 5 μM KC-A, and then cultured for 24 h in the absence or presence of 1 nM DHT or 10 nM Adione (**B**–**D**), 1 nM 11KDHT or 10 nM 11KAdione (**E**–**G**). The mRNA expression levels were analyzed by RT-qPCR analysis. ** *p* < 0.01, * *p* < 0.05, *NS p* > 0.05 denotes significantly different from the cells treated with DMSO alone. ^##^ *p* < 0.01, ^#^ *p* < 0.05, *NS p* > 0.05 denotes significantly different from the cells treated with 1 nM DHT, 10 nM Adione, 1 nM 11KDHT or 11KAdione alone.

Previously, several inhibitors of DHRS11 have been reported in its characterization studies [26,32]. We found that a structurally novel polyphenol KC-A showed potent DHRS11 inhibitory activity (IC_50_ = 0.35 ± 0.03 μM) comparable to the known inhibitors, carbenoxolone and luteolin [32]. Then, we examined the effect of KC-A on androgen signaling in the three PC cells (Figure 5). DHRS11 converts Adione and 11KAdione to the active androgens DHT and 11KDHT, as mentioned above. KC-A significantly decreased the expression of mRNAs for PSA and TMPRSS2 induced by Adione and 11KAdione in the cells. Surprisingly, KC-A also had significant inhibitory potency against the androgen signaling induced by DHT and 11KDHT. These results suggest a possibility that KC-A suppresses androgen signaling through other mechanisms in addition to DHRS11 inhibition.

**Figure 6:**
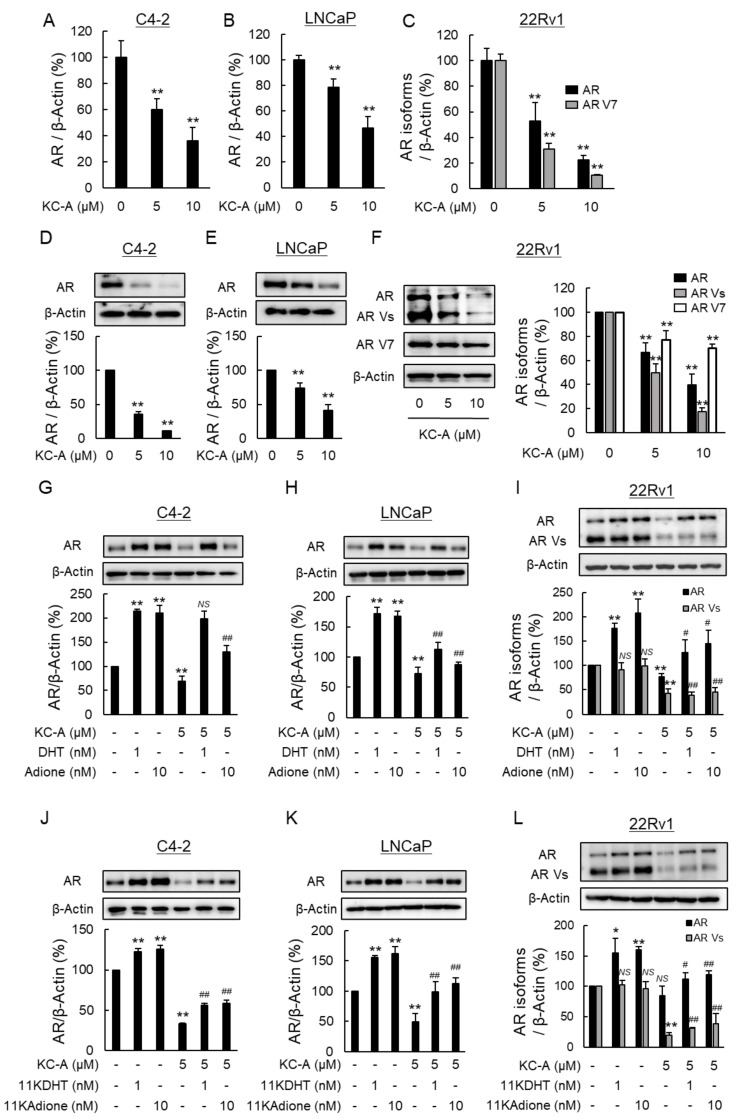
Effects of KC-A on AR expression in C4-2, LNCaP and 22Rv1 cells. (**A**–**C**) mRNA expression. The cells were treated for 24 h with the indicated concentrations of KC-A. The expression levels of the mRNAs for AR and AR V7 were analyzed by RT-qPCR. (**D**–**F**) AR protein expression. After 24 h-treatment of KC-A, the levels of AR, AR Vs and AR V7 in C4-2 (**D**), LNCaP (**E**) and 22Rv1 (**F**) cells were analyzed by immunoblotting ** *p* < 0.01 denotes significantly different from the cells treated with DMSO alone. (**G**–**L**) Effects of KC-A on AR expression induced by androgens. The cells were pretreated for 2 h without or with 5 μM KC-A, and then cultured for 24 h in the absence or presence of 1 nM DHT, 10 nM Adione (**G**–**I**), 1 nM 11KDHT or 10 nM 11KAdione (**J**–**L**). The protein expression levels of AR and AR Vs were analyzed by immunoblotting ** *p* < 0.01, * *p* < 0.05, *NS p* > 0.05 denotes significantly different from the cells treated with DMSO alone. ^##^ *p* < 0.01, ^#^ *p* < 0.05, *NS p* > 0.05 denotes significantly different from the cells treated with DHT, Adione, 11KDHT or 11KAdione alone.

We postulated an antagonistic action against AR as a possible action of KC-A leading to the suppression of DHT- or 11KDHT-induced androgen signaling and performed a reporter assay using AR-EcoScreen GR KO cells [33]. Contrary to expectation, KC-A did not inhibit the increased luciferase activity by DHT, Adione, 11KDHT or 11KAdione, which suggested that it does not exhibit AR antagonistic activity (Figure S2). Next, we examined the effect of KC-A on the expression of AR. In the absence of androgens, the treatment of C4-2, LNCaP, and 22Rv1 cells with KC-A significantly decreased the expression of mRNA for AR (Figure 6A–C). In 22Rv1 cells, the mRNA for a splicing variant of AR (AR V7) was also greatly decreased (Figure 6C). The decreases in AR and AR V7 were confirmed at protein levels in these cells (Figure 6D–F). So far, at least 17 AR Vs have been identified [34]. Among them, AR V7, which lacks the ligand binding domain, is homeostatically activated and is not regulated by CRPC drugs that target androgen signaling, and its upregulation is considered to be one of the causes of CRPC drug resistance [35]. Then, controlling the expression of both wild-type AR and AR V7 is an important strategy for the treatment of CRPC. Next, we examined the effect of KC-A on AR expression in the presence of androgens (Figure 6G–L). Since the binding of androgens, such as T and DHT, to AR increases the protein stability of AR [36,37], the AR protein expression levels may be linked to the extent of the androgen signaling. KC-A treatment significantly decreased AR protein expression levels in C4-2, LNCaP, and 22Rv1 cells, and also decreased the protein expression of AR Vs including AR V7 more than that of AR. These results suggest that KC-A has the potential to suppress androgen signaling through both DHRS11 inhibition and a decrease in AR and AR V7 expressions. Future works are needed to synthesize KC-A derivatives and investigate their structure-activity relationships, which will contribute to the search and/or development of compounds that exhibit more potent inhibitory activity for AR expression and/or androgen signaling.

**Figure 7:**
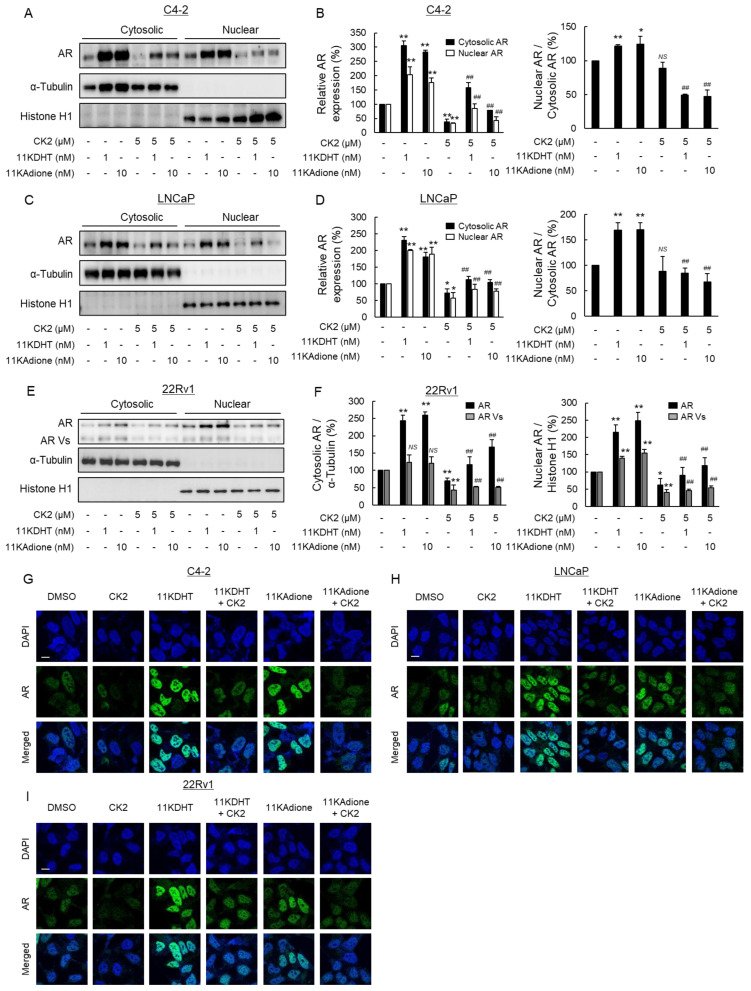
Effects of KC-A on 11-ketoandrogen-induced AR signaling in C4-2, LNCaP and 22Rv1 cells. The cells were pretreated for 2 h without or with 5 μM KC-A, and then cultured for 24 h in the absence or presence of 1 nM 11KDHT or 10 nM 11KAdione in the cells. (**A**–**F**) Nuclear expression of AR and AR Vs. The nuclei of the cells were fractionated from the cytosol by using a LysoPure Nuclear and Cytoplasmic Extractor Kit and analyzed by immunoblotting. The protein levels are expressed as a percentage of the band density relative to those in the control cells treated with DMSO alone. ** *p* < 0.01, * *p* < 0.05, *NS p* > 0.05 denotes significantly different from the cells treated with DMSO alone. ^##^ *p* < 0.01 denotes significantly different from the cells treated with 1 nM 11KDHT or 10 nM 11KAdione alone. (**G**–**I**) Immunofluorescence staining. The C4-2 (**G**), LNCaP (**H**) and 22Rv1 (**I**) cells were stained with the anti-AR antibody (green) and DAPI (blue). Merged images are also shown. Scale bar indicates 10 μm.

Since nuclear translocation of AR is essential for the activation of androgen signaling, we examined whether KC-A affects nuclear translocation in C4-2, LNCaP, and 22Rv1 cells treated with 11-ketoandrogens (Figure 7), DHT and Adione (Figure S3). The treatment with one of the androgens and their precursors apparently increased the cytosolic and nuclear AR levels in C4-2, LNCaP, and 22Rv1 cells. KC-A significantly decreased both the cytosolic and nuclear AR levels increased by the androgens, and it also reduced a ratio of nuclear AR expression to cytosolic AR. The decrease in nuclear AR was also observed by immunofluorescence staining (Figure 7G–I and Figure S3G–I). The data suggests that KC-A reduces nuclear AR expression not only through suppression of AR expression at the transcriptional level, but also through decreased production of potent androgens via DHRS11 inhibition in the cases treated with the androgen precursors (11-KAdione and Adione).

Although numerous natural compounds have been reported to have anticancer activity against PC cells, few natural compounds have been known to have antiandrogenic effects. In addition to the polyphenols listed above, stilbene was reported to inhibit androgen signaling via the inhibition of AR dimerization [38]. Other compounds, such as astaxanthin [39] and hinokitiol [40], also inhibit androgen-dependent cell proliferation in LNCaP cells, but the underlying mechanism remains unknown. Among these natural compounds with anti-androgenic activity, only luteolin is known to suppress the expression of mRNA for AR. Although luteolin decreases protein stability by inhibiting the association between AR and heat shock protein 90 [41], and downregulates AR V7 protein via the upregulation of miR-8080 [42], the downregulation mechanism remains unknown. Since KC-A decreased the expression of both AR protein and its mRNA, it must act at the transcriptional step, which may warrant further investigation for the elucidation of the mechanism.

### 2.4. Increased Sensitivity to Abi by KC-A

**Figure 8:**
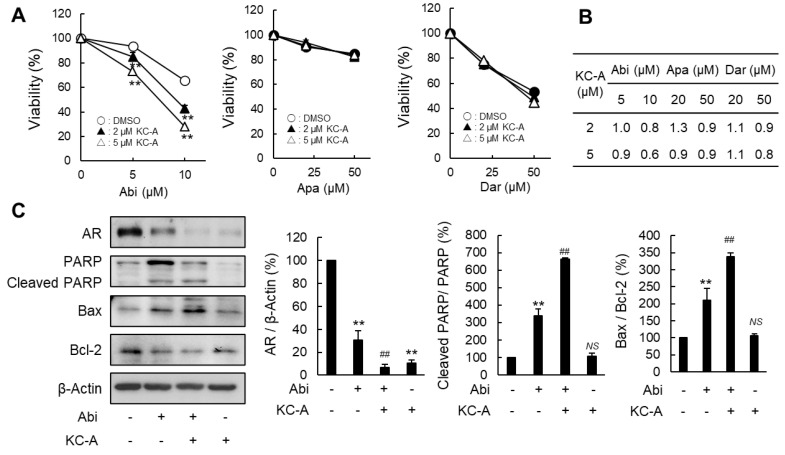

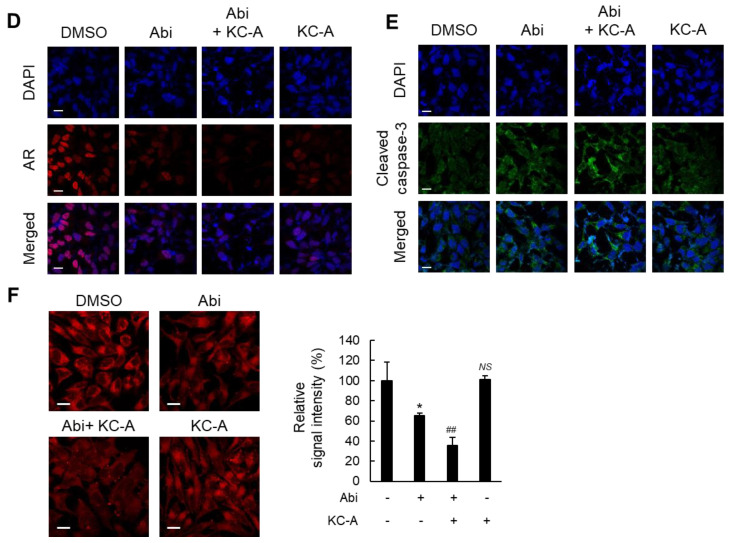
Effect of KC-A on Abi sensitivity in C4-2 cells. (**A**) Cell viability. The cells were treated with the indicated concentrations of CRPC drugs, Abi, apalutamide (Apa) or darolutamide (Dar) for 72 h in the absence or presence of indicated concentrations of KC-A and the cell viability was measured with an Alamar blue assay. ** *p* < 0.01 denotes significantly different from the cells treated with Abi alone. (**B**) Combined effects. Combination index (CI) values of the combination treatment were calculated by the CompuSyn software. CI < 1, CI = 1, and CI > 1 denotes synergism, additivity and antagonism effects, respectively. (**C**–**F**) The cells were treated without or with 10 μM Abi for 24 h in the absence or presence of 5 μM KC-A. (**C**) Protein expression of AR and apoptosis-associated proteins. The expression of AR, total and cleaved PARP, Bax, Bcl-2 and β-actin in the cell extracts were analyzed by immunoblotting. The ratio of AR/β-actin, PARP/cleaved PARP or Bax/Bcl-2 is expressed as a percentage of that in the control cells treated with DMSO alone. ** *p* < 0.01, *NS p* > 0.05 compared with the control cells. ^##^ *p* < 0.01 denotes significantly different from the cells treated with Abi alone. (**D**,**E**) Immunofluorescence staining. The cells were stained with DAPI (blue) and antibodies against AR (red, (**D**)), cleaved caspase-3 antibody (green, (**E**)). Merged images are also shown. Scale bar represents 20 μm. (**F**) MT-1 staining. Mitochondrial membrane potential of the cells was determined with a fluorescence probe MT-1. The fluorescence intensity was quantified using ImageJ, and the signal intensity in the treated cells is expressed as the percentage of that in the control cells treated with vehicle DMSO alone * *p* < 0.05, *NS p* > 0.05 compared with the control cells. ^##^ *p* < 0.01 denotes significantly different from the cells treated with Abi alone. Scale bar represents 20 μm.

Abi inhibits androgen signaling through suppressing androgen biosynthesis by CYP17A1 inhibition, and thereby leads to suppression of PC growth and a reduction in PSA level [43,44,45]. DHRS11 converts Adione and 11KAdione, which are synthesized by CYP17A1, into the more potent androgens, DHT and 11KDHT, respectively. Since, KC-A exhibited the dual action of DHRS11 inhibition and AR downregulation, we investigated whether KC-A enhances the anticancer activity of Abi using C4-2 cells. The anticancer activity of Abi was evident by its dose-dependent viability loss, which was markedly enhanced by the combination of KC-A. KC-A did not influence viability loss by the androgen receptor antagonists apalutamide (Apa) or darolutamide (Dar) (Figure 8A). Under the cotreatment with 5 μM KC-A and 10 μM Abi, the combination index (CI) was 0.6, which indicates the combination showed a synergistic effect (Figure 8B). On the other hand, CI values under the cotreatment of Apa or Dar with KC-A were about 1.0, indicating no additive or synergistic effect. This might be because KC-A acts on the same target as Apa and Dar, albeit with different mechanisms of action. Similar to previous reports [46], the treatment with Abi decreased AR protein levels via the inhibition of androgen synthesis in C4-2 cells, but the effect was less than that of KC-A (Figure 8C,D). Moreover, cotreatment with Abi and KC-A almost completely inhibited AR expression. The cotreatment also increased the ratio of cleaved PARP to total PARP compared to the treatment with Abi alone, suggesting that KC-A enhances Abi-induced apoptosis (Figure 8C,E). Since Abi is known to induce apoptosis in PC cells via mitochondrial dysfunction [45], we further examined the effect of Abi on mitochondrial function. The treatment with Abi alone increased the Bax/Bcl-2 ratio, while KC-A did not. Notably, the combination with Abi and KC-A significantly increased the Bax/Bcl-2 ratio (Figure 8C) and decreased the mitochondrial membrane potential compared to Abi alone (Figure 8F). In a mouse model with androgen deprivation therapy, the expression level of glucose transporter type 4, a marker of mitochondrial function, was shown to be decreased [47]. Therefore, the KC-A-mediated suppression of androgen signaling may enhance Abi-induced apoptotic cell death via the induction of mitochondrial dysfunction.

## 3. Materials and Methods

### 3.1. Chemicals and Reagents

(*E*)-4-Methoxycinnamaldehyde (CAS RN: 24680-50-0), iodine (CAS RN: 7553-56-2) and boron tribromide (CAS RN: 10294-33-4) were purchased from TCI (Tokyo, Japan). 4′,6′-Dimethoxy-2′-hydroxy-3-methyl acetophenone (CAS RN: 23121-32-6) was obtained from Carbosynth (Berkshire, UK). EtOH, DCM, DMSO, EtOAc and CHCl_3_ were purchased from KANTO KAGAKU (Tokyo, Japan). DHT was purchased from TCI, and Adione, 11KDHT and 11KAdione were from Steraloids (Newport, RI, USA). Abi and carbenoxolone were obtained from ChemScene (Monmouth Junction, NJ, USA) and Sigma-Aldrich (Saint Louis, MO, USA), respectively. All other chemicals were of the highest grade that could be obtained commercially.

### 3.2. General Experimental Procedures in Chemistry

IR spectra were recorded on a Perkin Elmer Spectrum 100 FTIR spectrometer (Waltham, MA, USA) and are reported in terms of frequency of absorption (cm^–1^). Optical rotations were measured on a P-1020 (JASCO, Tokyo, Japan) polarimeter equipped with a sodium lamp (589 nm). UV spectra were recorded on a UV-3600 (Shimazu, Kyoto, Japan) spectrophotometer. NMR experiments were performed on a JEOL ECA-500 spectrometer (Tokyo, Japan) at 298 K. All the 2D NMR spectra were acquired in DMSO-*d*_6_. Standard pulse sequences and phase cycling were used for DQF-COSY, HMQC, HMBC experiments. HRESIMS were acquired in negative ion mode on an IT-TOF spectrometer (Shimazu). Column chromatographies were performed over silica gel 60 (70–230 mesh, Merck, Darmstadt, Germany) and Sephadex LH-20 (GE Healthcare, Buckinghamshire, UK). Flash column chromatography was performed with Kanto silica gel 60N (Spherical, Neutral, 40–50 mm). Vacuum column chromatography was performed with silica gel 60H (Merck). HPLC separations were carried out using a Cosmosil 5C18-MS-II column (10 mm × 250 mm Nakarai tesque, INC (Kyoto, Japan), flow rate 4.0 mL/min) and a Shimazu 6AD series pumping system equipped with a Shimazu SPD-10AV index detector. TLC was conducted on silica 60 F_254_ gel-coated glass sheets (Merck).

### 3.3. Extraction and Isolation of KC-A

Flower parts of *Corex kobomugi* (3.1 kg fresh weight) collected in Tahara City, Aichi Prefecture, Japan, were used as the experimental material for this study. The fresh flower parts were extracted with methanol at room temperature, and the resulting extract was concentrated under reduced pressure to prepare a methanol extract (172 g). After suspending the methanol extract in water, liquid partitioning was performed with ethyl acetate and n-butanol to obtain the ethyl acetate fraction (15 g), the *n*-butanol fraction (24 g), and the water fraction (133 g), respectively. The ethyl acetate fraction was coarsely fractionated into Fr. 1~10 using Si CC in a chloroform and methanol mixture. Fr. 5 was then purified by Sephadex LH-20 (MeOH) CC and fractionated into six fractions (Fr. 5-1~5-6). Of these, Fr. 5-4 was separated by semi-preparative HPLC (40% MeCN 0.1% TFA) to yield KC-A (10.6 mg, t*R* = 11.38 min). The structure of KC-A was determined by a detailed analysis of spectral data from 1D and 2D NMR, as well as MS (See Figure S4).

**KC-A: 5,7-dihydroxy-8-methyl-2-[2-(4-hydroxyphenyl)ethenyl]-4*H*-1-benzopyran-4-one**

A pale yellow amorphous; UV (MeOH) λ_max_ (log ε) nm: 369 (3.80), 278 (3.46), 232 (3.56), 207 (3.70); IR ν_max_ cm^−1^: 2923, 2853, 1657, 1605, 1572, 1510, 1419, 1387, 1358, 1329, 1269, 1165, 1122, 1093, 963, 837, 800, 762 cm^−1^; ^1^H NMR (500 MHz, DMSO-*d*_6_) 12.84 (s, 5-OH), 10.84 (s, 4′-OH), 10.04 (brs, 7-OH), 7.59 (brd, *J* = 6.4 Hz, H-2′,6′), 7.54 (d, *J* = 15.5 Hz, H-12), 6.97 (brd, *J* = 15.5 Hz, H-11), 6.84 (brd, *J* = 6.4 Hz, H-3′,5′), 6.28* (s, H-3), 6.28* (s, H-6), 2.21 (s, 8-Me), *overlapping; ^13^C NMR (125 MHz, DMSO-*d*_6_) δ 182.0 (C-4), 162.8 (C-2), 162.0 (C-7), 159.5 (C-4′), 158.9 (C-5), 154.5 (C-9), 137.0 (C-12), 129.9 (C-2′,6′), 126.0 (C-1′), 116.7 (C-11), 115.9 (C-3′,5′), 106.8 (C-3), 103.8 (C-10), 101.9 (C-8), 98.0 (C-6), 7.6 (C-8-Me); HR-ESI-IT-TOFMS: *m*/*z* 309.0753 ([M − H]^−^, Calcd. C_18_H_13_O_5_: 309.0763).

### 3.4. Synthesis of KC-A

**Scheme 1:**
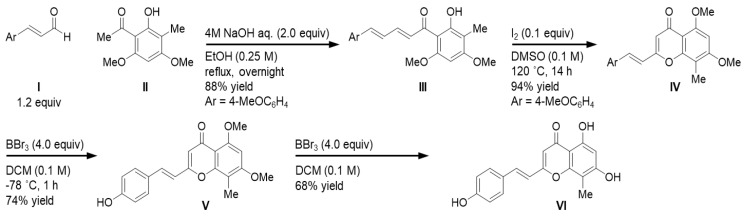
Synthesis of KC-A.

KC-A was prepared as illustrated in Scheme 1.

**1-(2-Hydroxy-4,6-dimethoxy-3-methylphenyl)-5-(4-methoxyphenyl)-2,4-pentadien-1-one (III)**

A solution of (E)-4-methoxycinnamaldehyde **I** (1.96 g, 12 mmol) and 4′,6-dimethoxy-2′-hydroxy-3-methyl acetophenone **II** (2.10 g, 10 mmol) in EtOH (40 mL) was stirred at 60 °C. 4M NaOH aq. (5 mL) was added into the resulting solution, and the mixture was refluxed, with stirring, overnight. The resulting solution was cooled to room temperature and concentrated in vacuo. The residue was diluted with water (20 mL) and extracted with EtOAc (30 mL × 3). The combined organic layer was washed with brine (20 mL). The organic phase was dried over MgSO4, filtered and concentrated in vacuo. The obtained crude mixture was recrystallized from (EtOAc/nHex) to furnish a title compound **III** as a mixture of E/Z isomers (88%, 3.12 g), and used for the next step without further purifications.

**5,7-Dimethoxy-8-methyl-2-[2-(4-methoxyphenylethenyl)]-4*H*-1-benzopyran-4-one (IV)**

A solution of **III** (1.06 g, 1.0 equiv, 3 mmol) and I_2_ (76.2 mg, 0.1 equiv, 0.3 mmol) in DMSO (20 mL) was stirred at 120 °C for 20 h. The resulting solution was cooled to room temperature and diluted with Ethyl acetate (20 mL) and washed with water (20 mL × 2). The organic layer was washed with brine. The organic phase was dried over MgSO_4_, filtered and concentrated in vacuo. The resulting mixture was purified by flash column chromatography on silica gel (CHCl_3_:Methanol = 20:1) to give 993 mg of **IV** as a yellow powder (94% yield).

^1^H NMR (400 MHz, CDCl_3_) δ 7.51 (d, *J* = 8.8 Hz, 2H), 7.46 (d, J = 16.0 Hz, 2H), 6.92 (d, *J* = 8.8 Hz, 2H), 6.59 (d, *J* = 16.0 Hz, 1H), 6.39 (s, 1H), 6.13 (s, 1H), 3.98 (s, 3H), 3.95 (s, 3H), 3.85 (s, 3H), 2.31 (s, 3H) (See Figure S4).

**5,7-Dimethoxy-8-methyl-2-[2-(4-hydroxyphenylethenyl)]-4*H*-1-benzopyran-4-one (V)**

A solution of **IV** (573 mg, 1.0 equiv, 1.63 mmol) in DCM (16.3 mL) was cooled to −78 °C, with stirring, under Ar atmosphere. BBr_3_ (1 M in DCM, 6.52 mL, 4 equiv, 6.52 mmol) was added dropwise into a solution, and stirred for 1 h at −78 °C. The resulting mixture was quenched with NaHCO_3_ aq. (16.3 mL) at −78 °C and stirred for 15 min at room temperature. The mixture was extracted with DCM (30 mL × 3). The combined organic phase was dried over MgSO_4_, filtered and concentrated in vacuo. The residue was powdered and washed with ether (10 mL) to obtain 412 mg of **V** (74%).

^1^H NMR (400 MHz, CDCl_3_) δ 7.59–7.53 (m, 3H), 6.95 (d, *J* = 9.2 Hz, 2H), 6.65 (d, *J* = 16.0 Hz, 1H), 6.40 (s, 1H), 6.14 (s, 1H), 3.92 (s, 3H), 3.88 (s, 3H), 2.92 (s, 3H) (See Figure S4).

**5,7-Dihydroxy-8-methyl-2-[2-(4-hydroxyphenylethenyl)]-4*H*-1-benzopyran-4-one (KC-A, VI)**

A solution of 5 (67.6 mg, 0.2 mmol) and BBr3 (1M in DCM, 0.8 mL, mousu0.8 mmol, 8.0 equiv) in DCM (2 mL) was stirred, at room temperature, overnight. The resulting solution was concentrated in vacuo. The residue was purified by vacuum column chromatography on silica gel to obtain 44.6 mg of KC-A (68% yield).

^1^H NMR (500 MHz, DMSO-*d*_6_) 12.85 (s, 5-OH), 7.59 (d, *J* = 8.3 Hz, H-2′,6′), 7.54 (d, *J* = 16.2 Hz, H-12), 6.97 (d, *J* = 16.2 Hz, H-11), 6.84 (d, *J* = 8.3 Hz, H-3′,5′), 6.29 (s, H-3), 6.28 (s, H-6), 2.21 (s, 8-Me); ^13^C NMR (125 MHz, DMSO-*d*_6_) δ 182.0 (C-4), 162.8 (C-2), 162.0 (C-7), 159.5 (C-4′), 158.9 (C-5), 154.5 (C-9), 137.0 (C-12), 129.9 (C-2′,6′), 126.0 (C-1′), 116.7 (C-11), 115.9 (C-3′,5′), 106.8 (C-3), 103.8 (C-10), 101.9 (C-8), 98.0 (C-6), 7.6 (C-8-Me); HR-ESI-IT-TOFMS: *m*/*z* 309.0758 ([M − H]^−^, Calcd. C_18_H_13_O_5_: 309.0763) (See Figure S4).

### 3.5. Enzyme Activity Assay

Homogenous recombinant DHRS11 were prepared as previously described [26]. The reductase activity of DHRS11 was determined at 25 °C by measuring the rate of change in NADPH absorbance (at 340 nm). The IC_50_ values for the inhibitors were determined in the reaction mixture that consisted of 0.1 M potassium phosphate, pH 7.4, 0.1 mM NADPH, 0.1 mM isatin, enzyme, and/or an inhibitor in a total volume of 2.0 mL. The IC_50_ values are expressed as the means ± SDs of at least three determinations.

### 3.6. Cell Culture and Transfection

PC 22Rv1, LNCaP and C4-2 cells were obtained from the American Type Culture Collection (Manassas, VA, USA), and cultured in RPMI-1640 medium (Fujifilm Wako Pure Chemical, Osaka, Japan) supplemented with 5% fetal bovine serum (FBS, Sigma-Aldrich, St. Louis, MO, USA), 10 mM HEPES (pH 7.0), penicillin (100 U/mL) and streptomycin (100 µg/mL) at 37 °C in a humidified incubator containing 5% CO_2_. In the experiments treated with androgens, after the cells were incubated for 24 h, the medium was changed to a medium supplemented with 2% charcoal-stripped fetal bovine serum (CS-FBS, Biological Industries, Kibbutz Beit-Haemek, Israel) and then treated for the indicated period with steroids and/or agents, which were dissolved in dimethyl sulfoxide (DMSO). The cells were transfected with siRNA against DHRS11, AKR1C3 or universal negative control siRNA (Japan Bio Services, Saitama, Japan; Table S1), using a Lipofectamine RNAi MAX reagent (Thermo Fisher Scientific, Waltham, MA, USA) according to the manufacturer’s instructions.

### 3.7. Cell Viability

The cells were plated into a 96-well microplate at a density of 1 × 10^4^ cells/well in the growth medium supplemented with 2% FBS on the day before the experiment was performed. Following treatment with agents for 72 h, cell viability was evaluated by an Alamar blue assay using resazurin [48].

### 3.8. RNA Extraction and Reverse Transcription-Quantitative Polymerase Chain Reaction (RT-qPCR)

Total RNA was extracted with TRI reagent (Molecular Research Center, Cincinnati, OH, USA) and reverse-transcribed with a ReverTra Ace qPCR RT Kit (Toyobo, Osaka, Japan) according to the manufacturer’s instructions. qPCR was performed with THUNDERBIRD SYBR qPCR Mix (Toyobo) with a CFX96 Touch Deep Well Real-Time PCR Detection System (Bio-Rad Laboratories, Hercules, CA, USA). The sequences of primers used are shown in Table S2.

### 3.9. Western Blotting

The cells were washed twice with Dulbecco’s phosphate-buffered saline and suspended in 10 mM Tris-HCl, pH 8.0, containing 50 mM sodium phosphate and 8 M urea, and then homogenized by sonication. The nuclear and cytosolic fractions of the cells were divided with a LysoPure Nuclear and Cytoplasmic Extractor Kit (Fujifilm Wako Chemicals) as previously described [49]. The protein concentration was determined using a bicinchoninic acid protein assay kit (Nacalai Tesque, Kyoto, Japan). The cell lysates were subjected to Western blotting analysis, which was performed as described previously [50]. Primary antibodies used in Western blotting analysis were listed in Table S2. The immunoreactive proteins were visualized using horseradish peroxidase-conjugated secondary antibodies and an enhanced chemiluminescence substrate system (GE Healthcare). The band density was quantified with ImageJ software (National Institute of Health, Bethesda, MD, USA).

### 3.10. Immunofluorescence Staining and MT-1 Staining

Immunofluorescence staining was performed as described previously [51]. Briefly, the cells were cultured on poly-D-lysine-coated cover glasses, washed with DPBS, fixed with 4% paraformaldehyde for 10 min, and then permeabilized with DPBS supplemented with 0.1% Triton-X100 and 100 mM glycine for 10 min. After blocking with DPBS containing 0.1% Tween 20 and 1% bovine serum albumin for 30 min, the cells were incubated with a diluted primary antibody (1:500) against AR, Ki67 or cleaved caspase-3 for 1 h at room temperature. After washing with DPBS containing 0.1% Tween 20 three times, the samples were then exposed to the Alexa Fluor 488-conjugated secondary antibody or Alexa Fluor 555-conjugated secondary antibody (1:500, Thermo Fisher Scientific, Waltham, MA, USA) for 1 h. The cells were mounted with DAPI-Fluoromount-G (Southern Biotech, SBA, Birmingham, AL, USA) and the fluorescence signals were examined with a laser confocal microscope Zeiss LSM-700 (Carl Zeiss, Jena, Germany). For monitoring of the mitochondrial membrane potential, the cells were incubated with 5 μM MT-1 probe for 30 min. The other conditions were the same as above.

### 3.11. AR-EcoScreen Reporter Gene Assay

An AR-EcoScreen cell line with functional knockout of the glucocorticoid receptor (AR-EcoScreen GR KO cells) was obtained from the Japanese Collection of Research Bioresources Cell Bank (Osaka, Japan) [33]. The cells were propagated in RPMI-1640 medium supplemented with 5% FBS, zeocin (200 µg/mL), hygromycin (100 µg/mL) as well as penicillin (100 U/mL) and streptomycin (100 µg/mL). For the assay, the cells were cultured in phenol red-free RPMI-1640 medium supplemented with 2% CS-FBS. The androgen response element-mediated transactivation of a luciferase reporter gene was determined in the cells using the Steady-Glo luciferase assay system (Promega, Madison, WI, USA) following the manufacturer’s instructions. Luminescence was measured using the GloMax-Multi Detection System (Promega, Madison, WI, USA), and the measurement of viable cells via Alamar blue assay was used to standardize the luciferase luminescence.

### 3.12. Statistical Analysis

Data are expressed as the means ± SDs of at least three independent experiments. Statistical analyses were performed using ystat2018.xls for Windows (Igakutosyosyuppan co., Ltd., Tokyo, Japan). One-way ANOVA followed by Bonferroni’s post-hoc comparisons tests were used for multiple comparisons. Comparison of the two groups was performed using paired Student’s *t*-test. A *p* value < 0.05 was considered statistically significant.

## 4. Conclusions

The present study has revealed that 11-oxygenated androgens, as well as classical androgens, activate androgen signaling in PC cells. Because DHT levels in castrated PC patients do not decrease completely, the intratumoral conversion of adrenal androgen precursors to active androgens has been shown to be involved in the development of CRPC. In the human adrenal gland, androgen precursors such as DHEA, DHEAS, A4 and 11OHA4 are pooled at high concentrations, and 11-oxygenated androgens are additional sources of intratumoral androgens. We have recently reported that DHRS11 exhibits 17βHSD activity, which is involved in intratumoral conversions, such as AKR1C3 and HSD17B3. The present study also demonstrates that DHRS11 is involved in the synthesis of active conventional androgens and 11-oxygenated androgens in PC cell lines. To elucidate the pathophysiological significance of DHRS11, it is desirable to investigate its expression in patients with CRPC in future work. Furthermore, we discovered KC-A as a novel DHRS11 inhibitor that is effective at the cellular level. Unexpectedly, KC-A also exerted a potent suppressive effect on AR expression. Therefore, KC-A suppressed androgen signaling in PC cells through both DHRS11 inhibition and AR downregulation. Additionally, KC-A was suggested to enhance the anticancer activity of Abi, a CRPC drug. Thus, KC-A might be a potential lead compound to develop drugs for the prevention and treatment of CRPC.

## Supplementary Materials

The following are available online at https://www.mdpi.com/article/10.3390/ijms232214356/s1.

## Abbreviations

11KA4	11-keto-4-androstene-3,17-dione
11KAdione	11-keto-5α-androstane-3,17-dione
11KDHT	11-keto-5α-dihydrotestosterone
11KT	11-ketotestosterone
11OHA4	11-hydroxy-4-androstene-3,17-dione
17βHSD	17β-hydroxysteroid dehydrogenase
A4	4-androstene-3,17-dione
Abi	abiraterone acetate
Adione	5α-androstane-3,17-dione
AKR	aldo-keto reductase
Apa	apalutamide
AR	androgen receptor
AR Vs	androgen receptor variants
CI	combination index
CRPC	castration-resistant prostate cancer
CS-FBS	charcoal-stripped fetal bovine serum
Dar	darolutamide
DHEA	dehydroepiandrosterone
DHEAS	dehydroepiandrosterone sulfate
DHRS11	dehydrogenase/reductase SDR family member 11
DHT	5α-dihydrotestosterone
DMSO	dimethyl sulfoxide
FBS	fetal bovine serum
HITCC	8-hydroxy-2-imino-*N*-(*p*-tolyl)-2*H*-chromene-3-carboxamide
PC	prostate cancer
PSA	prostate specific antigen
RT-qPCR	reverse transcription-quantitative polymerase chain reaction
T	testosterone
TMPRSS2	transmembrane protease serine 2
