# Supplementary material for: Antiandrogenic Effects of a Polyphenol in Carex kobomugi through Inhibition of Androgen Synthetic Pathway and Downregulation of Androgen Receptor in Prostate Cancer Cell Lines
Source: Int J Mol Sci. 2022 Nov 18;23(22):14356. doi: 10.3390/ijms232214356 (PMC9696374; doi:10.3390/ijms232214356)
Supplement: Supplementary file 1 [file ijms-23-14356-s001.zip › ijms-2009643 - Supplementary materials (proof check).pdf]

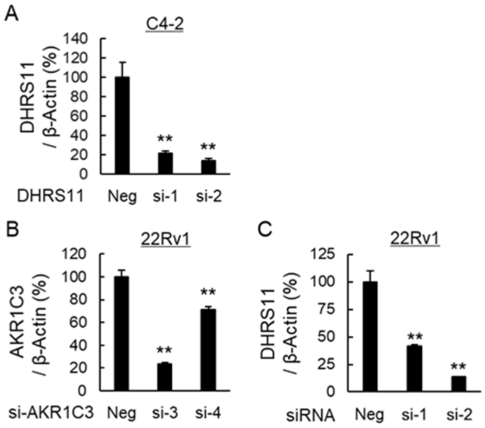

Figure S1. Knockdown efficiency of siRNA of DHRS11 and AKR1C3 in C4-2 (A) and 22Rv1 cells (B, C). The cells were transfected with universal negative siRNA (Neg), DHRS11 siRNA (si-1 and si-2) or AKR1C3 siRNA (si-3 and si-4) and cultured for 48h. The mRNA expression levels of DHRS11 and AKR1C3 in the cells were analyzed by RT-qPCR. **p < 0.01 significantly different from the cells treated with Neg.

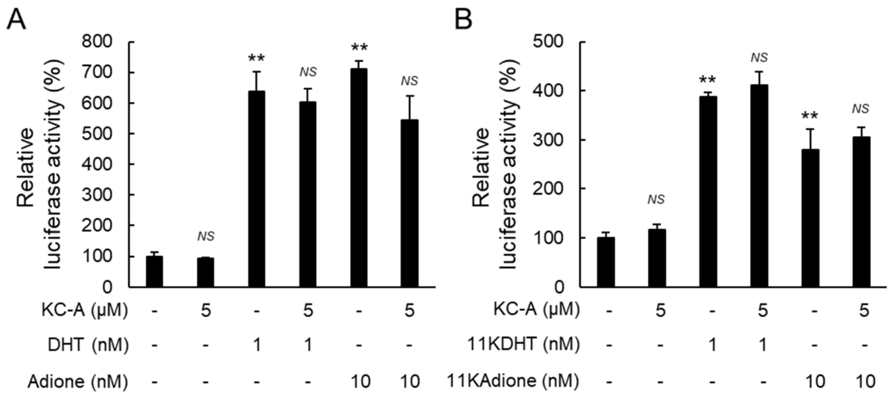

Figure S2. Luciferase activity in AR EcoScreen GR KO cells. The luciferase activities were evaluated by *in vitro* reporter gene assays using the AR-EcoScreen GR KO cells. Relative luciferase activity was presented as the relative ratio of firefly luciferase activity using Steady-Glo luciferase assay kit to the cell numbers calculated by alamar blue assay. The cells were pretreated for 2 h without or with 5 μM KC-A, and then cultured for 24 h in the absence or presence of 1 nM DHT, 10 nM Adione (A), 1 nM 11KDHT or 10 nM 11KAdione (B). **p < 0.01, *NS* p > 0.05, significantly different from the control cells treated with DMSO alone. *NS* p > 0.05, significantly different from the cells treated with DHT, Adione, 11KDHT or 11KAdione alone.

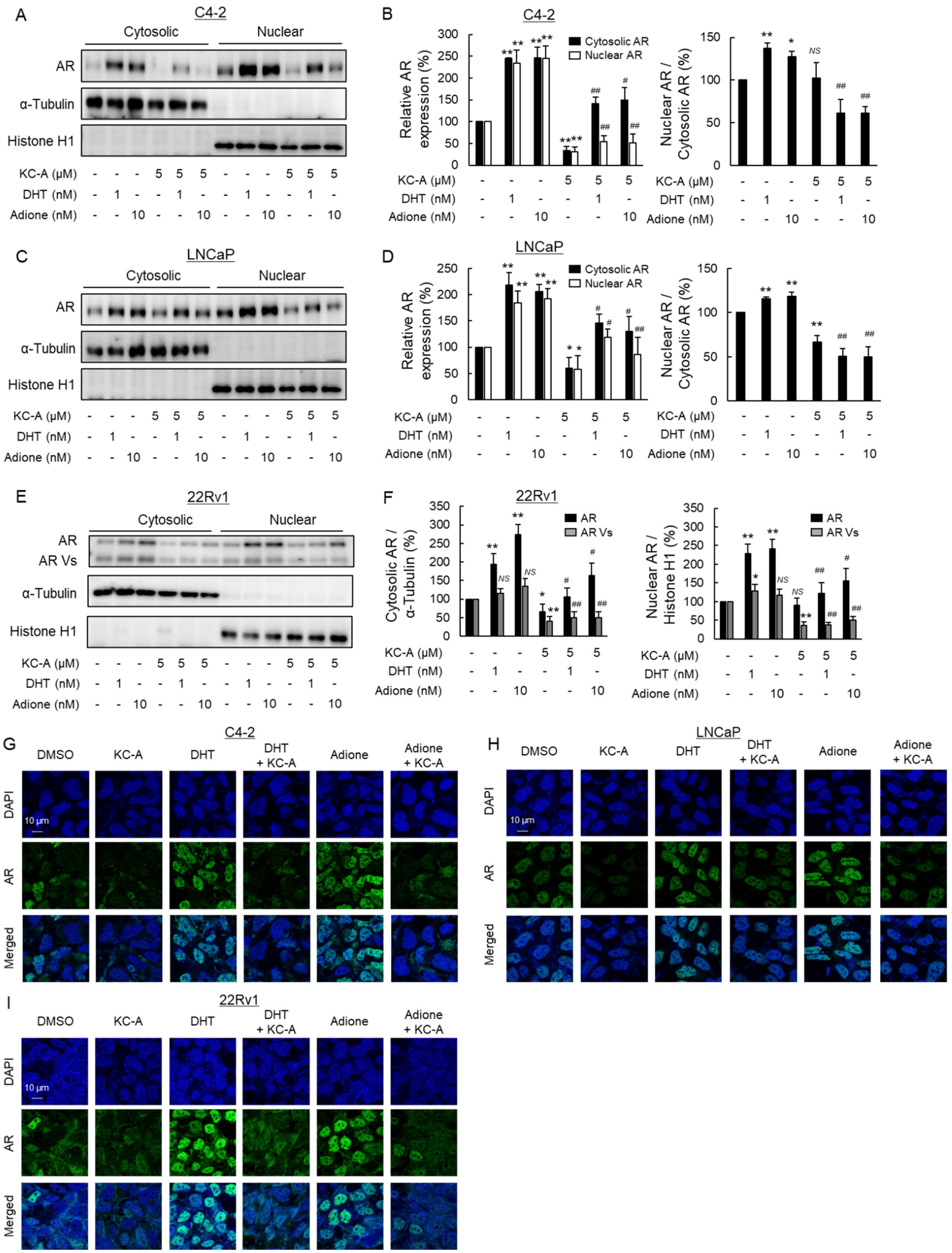
A
C4-2
Cytosolic
Nuclear
AR
α-Tubulin
Histone H1
KC-A (μM)
DHT (nM)
Adione (nM)
B
Relative AR expression (%)
Cytosolic AR
Nuclear AR
Nuclear AR / Cytosolic AR (%)
C
LNCaP
D
E
22Rv1
AR Vs
F
Cytosolic AR / α-Tubulin (%)
Nuclear AR / Histone H1 (%)
G
H
I
DMSO
KC-A
DHT
DHT + KC-A
Adione
Adione + KC-A
DAPI
Merged
10 μm

Figure **S3. Effects of KC-A on androgens-induced AR signaling in C4-2, LNCaP and 22Rv1 cells.** The cells were pretreated for 2 h without or with 5 μM KC-A, and then cultured for 24 h in the absence or presence of 1 nM DHT or 10 nM Adione in the cells. (A-F) Nuclear expression of AR and AR Vs. The nuclei of the cells were fractionated from the cytosol by using LysoPure Nuclear and Cytoplasmic Extractor Kit, as described in the Experimental Section and analyzed by immunoblotting. The protein levels are expressed as a percentage of the band density relative to those in the control cells treated with DMSO alone. $^{**}p < 0.01$, $^{*}p < 0.05$, *NS* $p > 0.05$ significantly different from the cells treated with DMSO alone. $^{\#\#}p < 0.01$, $^{\#}p < 0.05$ significantly different from the cells treated with 1 nM DHT or 10 nM Adione alone. (G-I) Immunofluorescence staining. The cells were stained with the anti-AR antibody (green) and DAPI (blue). Merged images were also shown. Scale bar indicates 10 μm.

Figure S4-1 The ^{1}H NMR spectrum of KC-A (1) in DMSO-d_6 (500 MHz)
Figure S4-2 The ^{13}C NMR spectrum of KC-A (1) in DMSO-d_6 (125MHz)
Figure S4-3 The DQF-COSY spectrum of KC-A (1) in DMSO-d_6
Figure S4-4 The HMQC spectrum of KC-A (1) in DMSO-d_6
Figure S4-5 The HMBC spectrum of KC-A (1) in DMSO-d_6
Figure S4-6 The IR spectrum of KC-A (1)
Figure S4-7 The UV spectrum of KC-A (1) in MeOH
Figure S4-8 The HR-ESI-MS spectrum of KC-A (1)

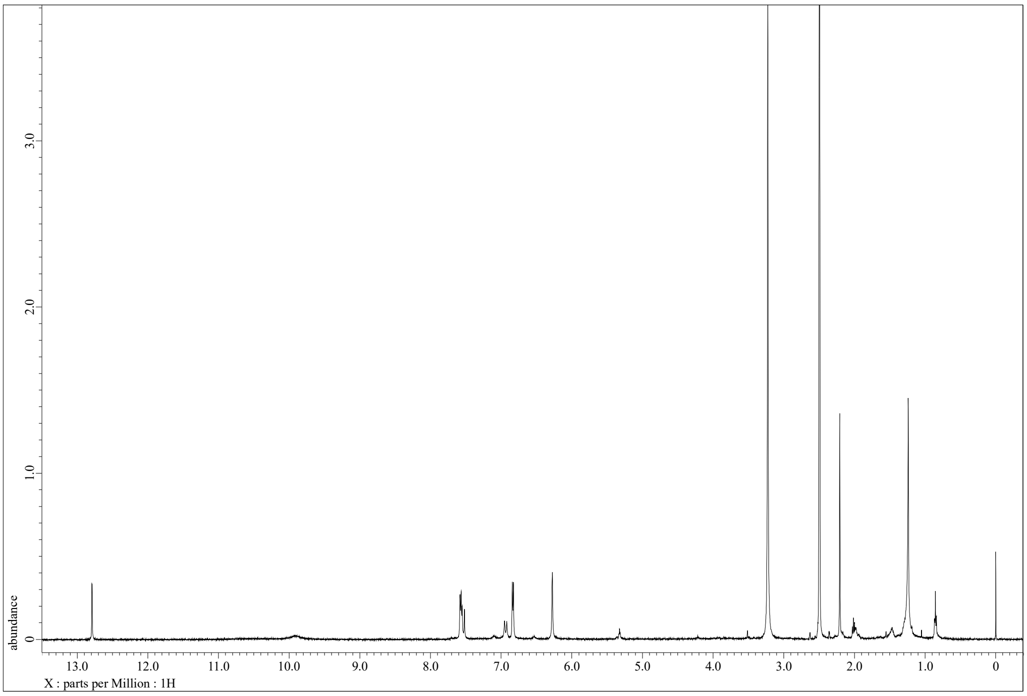

Figure S4-1 The ^{1}H NMR spectrum of KC-A in DMSO-d_6 (500 MHz)

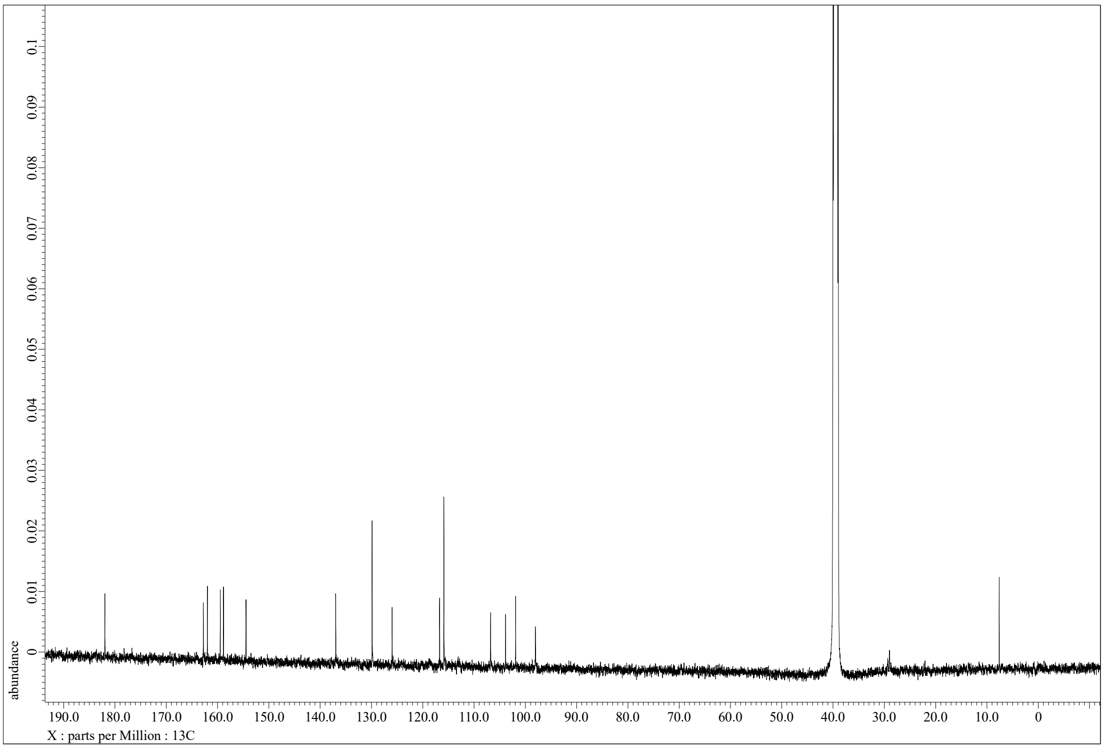

Figure S4-2 The ^{13}C NMR spectrum of KC-A in DMSO-d_6 (125MHz)

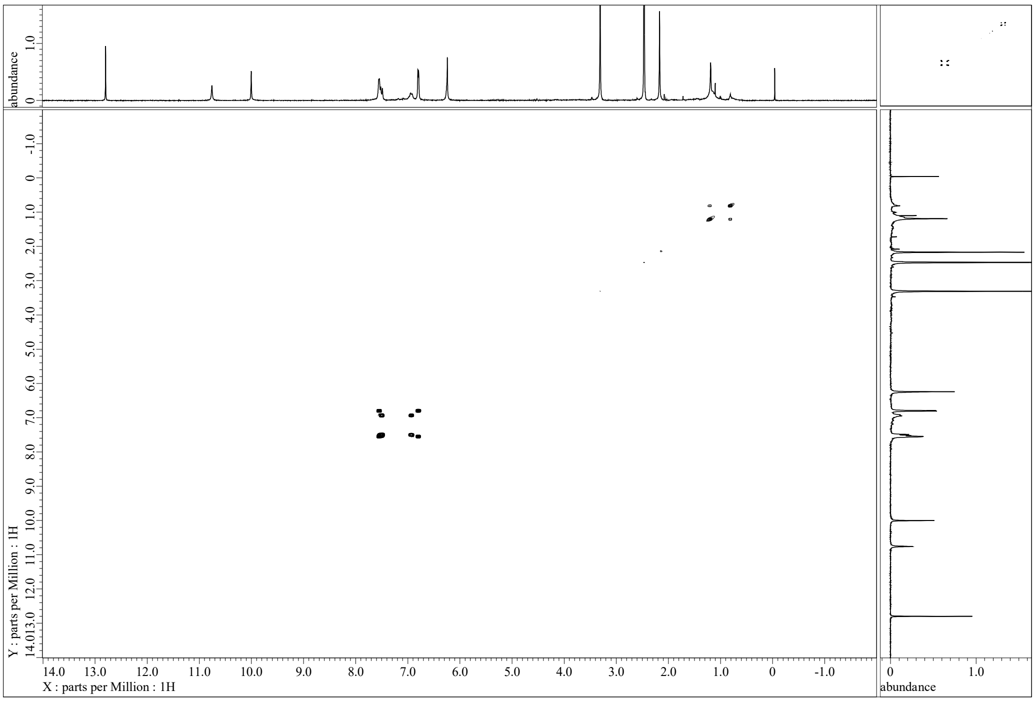

Figure S4-3 The DQF-COSY spectrum of KC-A in DMSO-d_6 (500 MHz)

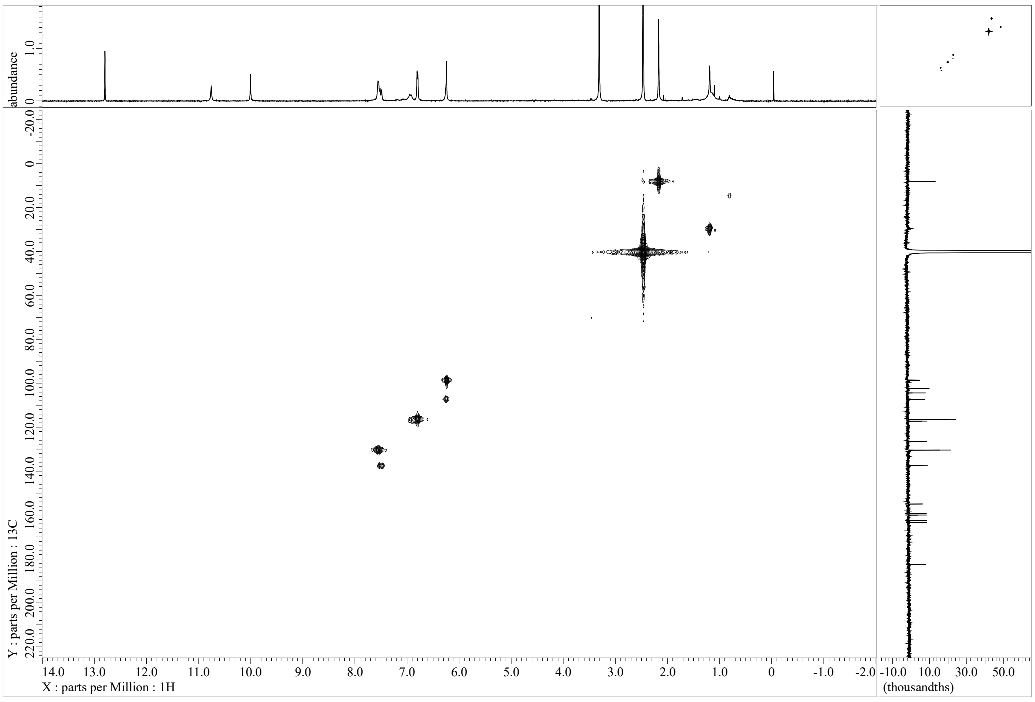

Figure S4-4 The HMQC spectrum of KC-A (**1**) in DMSO-d_6

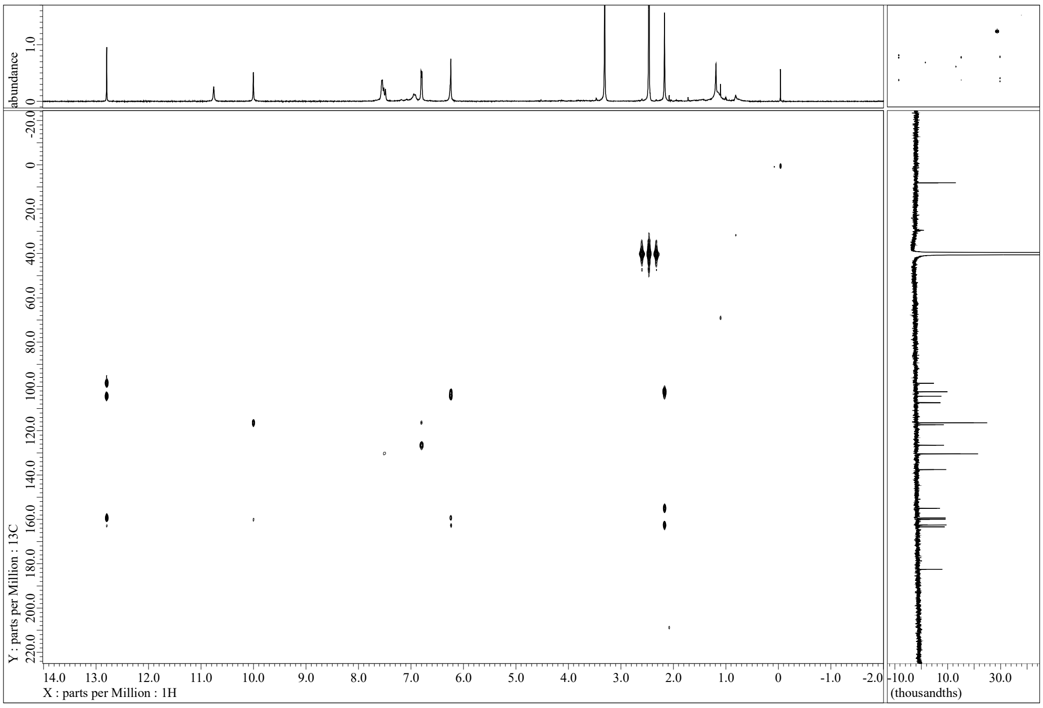

Figure S4-5 The HMBC spectrum of KC-A in DMSO-d_6

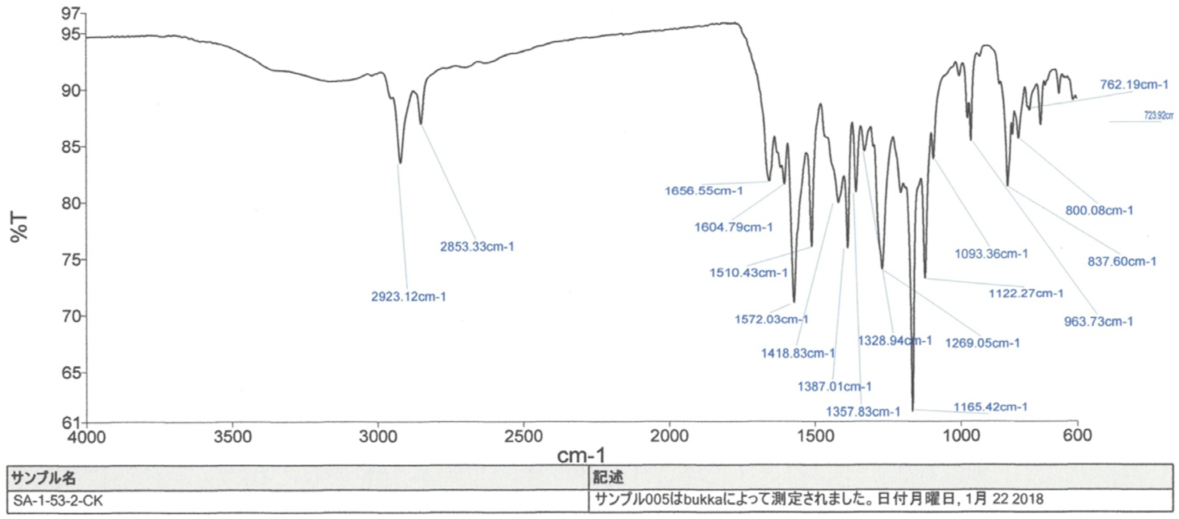

Figure S4-6 The IR spectrum of KC-A

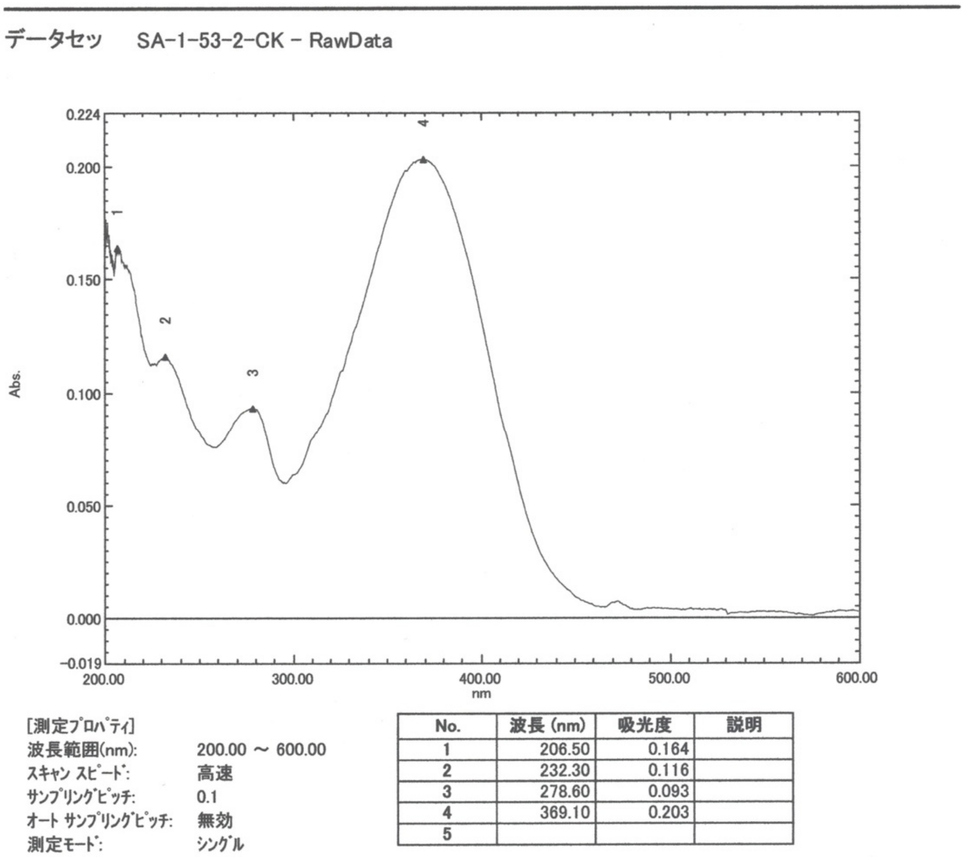

Figure S4-7 The UV spectrum of KC-A in MeOH

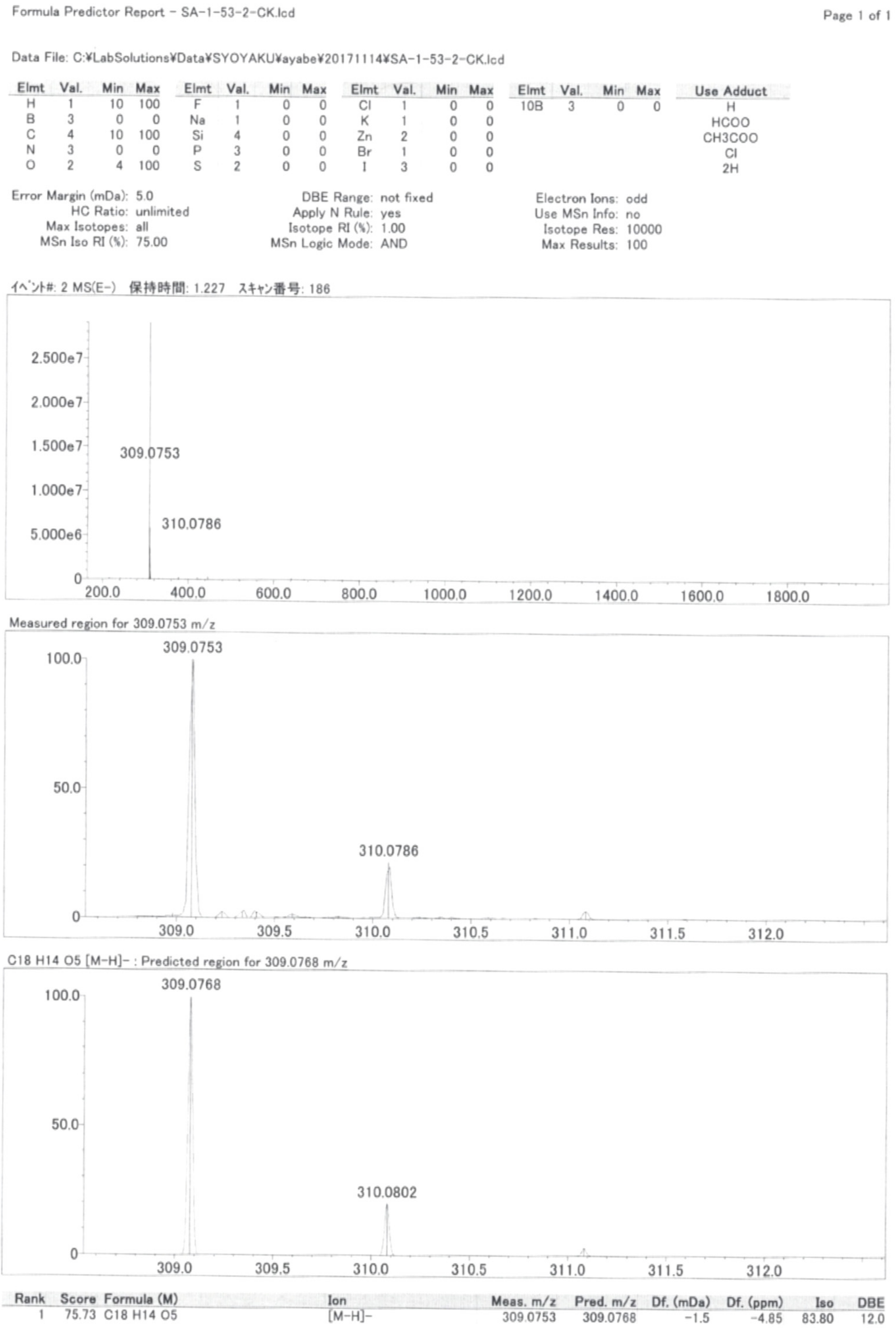

Figure S4-8 The HR-ESI-MS spectrum of KC-A

Table S1. Sequences of siRNAs against DHRS11 and AKR1C3.

	Sense (5′ --- 3′)	Antisense (5′ --- 3′)
Universal negative siRNA	UUCUCCGAACGUGUCACGUdTdT	ACGUGACACGUUCGGAGAAdTdT
DHRS11 siRNA-1 (si-1)	CACAAUUCGCCUUCAAACUdTdT	AGUUUGAAGGCGAAUUGUGdtdC
DHRS11 siRNA-2 (si-2)	CUUAUAUCUGUGUUGUUAUdTdT	AUAACAACACAGAUAUAAGdGdT
AKR1C3 siRNA-1 (si-3)	UCUCCACUAUUUUAACAGUdTdT	ACUGUUAAAAUAGUGGAGAdTdT
AKR1C3 siRNA-2 (si-4)	GGUGAGGAACUUUCACCAAdTdT	UUGGUGAAAGUUCCUCACCdTdT

Table S2. Primers used in the RT-qPCR analysis.

	Forward primer (5′ --- 3′)	Reverse promer (5′ --- 3′)
PSA	CGCAAGTTCACCCTCAGAAGGT	GACGTGATACCTTGAAGCACACC
TMPRSS2	CCTCTAACTGGTGTGATGGCGT	TGCCAGGACTTCCTCTGAGATG
AR	ATGGTGAGCAGAGTGCCCTATC	ATGGTCCCTGGCAGTCTCCAAA
AR V7	CCATCTTGTCGTCTTCGGAAATGTTA	TTTGAATGAGGCAAGTCAGCCTTTCT
DHRS11	CACCAGTGGTTGGAAGGACA	GTCGTGGAGTTTGAAGGCGA
AKR1C3	TCAAAGCTTTGGTCCACTTTTCA	CCATTTTCATCTGTTGGTGAAAG
β-Actin	CCACGAAACTACCTTCAAC	GATCTTCATTGTGCTGGG
